# Intralenticular subcapsular air

**DOI:** 10.4103/0301-4738.64145

**Published:** 2010

**Authors:** Sribhargava Natesh, Hima Bindu Adusumilli, Naresh Kumar Yadav, Priya B V

**Affiliations:** Department of Vitreo Retinal, Narayana Nethralaya, Bangalore, India

Dear Editor,

Lens injury has been reported as a complication after an intravitreal injection. Accidental lens touch, cataract formation, dislocation of lens and zonular tears are a possibility. Improper technique, inexperience of the surgeon, patient's head movement at the time of the injection can be the reasons.[1] In one of the major trials involving intravitreal injections, the rate of traumatic cataract was 0.7% (5 in 892).[2] We report an unusual case where intralenticular air was noted after an intravitreal injection of bevacizumab (Avastin, Medihauxe International Ltd). To the best of our knowledge, after a Pubmed search, no case with intralenticular subcapsular air following an intravitreal injection has been reported.

**Figure 1:**
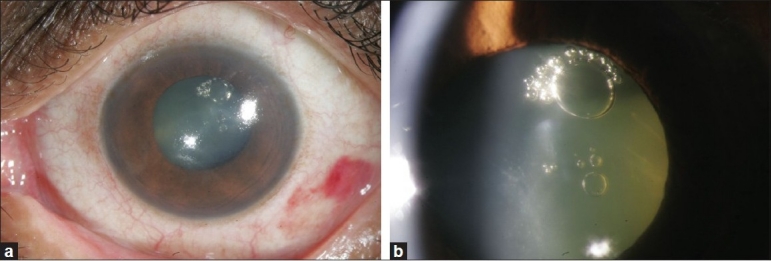
(a, b) Intralenticular subcapsular air

A 59-year-old diabetic male presented with complaints of floaters, of 15 days duration, in the left eye. His best corrected visual acuity was 20/30 and 20/40 in right and left eyes respectively. Anterior segment examination showed early posterior subcapsular cataract with cortical changes in both eyes. Fundus showed proliferative diabetic retinopathy (neovascularization elsewhere) in the right eye and diffuse vitreous hemorrhage in the left eye. He underwent panretinal photocoagulation in the right eye and intravitreal injection of bevacizumab in the left eye. Under topical anesthesia, intravitreal 0.05ml (1.25mg) bevacizumab was injected using a 30 gauge needle in the left eye, 3.5mm from the limbus in the inferotemporal quadrant. Nothing unusual was noted by the surgeon who had seen the drug enter the vitreous cavity. After the procedure, slit lamp examination of the left eye showed few pockets of air in the lens in the anterior sub capsular space superotemporally [Figs. 1a and b]. There was no evidence of any anterior or posterior capsular tears, opacification or dislocation of lens. There was no phacodonesis. Patient did not have any symptoms. Subsequently, there was no worsening of vision or cataract progression in the left eye. The intraocular pressure was within normal limits. Air pockets disappeared after two weeks. Patient subsequently underwent pan retinal photocoagulation in the left eye. His vision after one year of follow-up was 20/20 in the left eye with stable retinopathy. The cataract did not progress except for the sub capsular opacity in the area of previous air cells.

With the appropriate training of the surgeons in the technique, the lens complications expected after an intravitreal injection are minimal, but sudden head movements of the patient or accidental touch of the needle tip to the lens can never be avoided and even intralenticular injection of triamcinolone acetonide has been reported.[1,3,4] In this particular case, both the mechanism of the injury and the path of air entry into subcapsular space are difficult to explain.

A careful slit lamp examination was done to locate the needle track in the lens but except for the preexisting cortical changes and early posterior subcapsular cataract, there were no obvious lens changes. Nonprogressive, localised lenticular opacities, such as capsular scars, opacities along the track of the injury, or posterior subcapsular opacities have also been described following penetrating injuries with small sharp objects such as needles.[5] A clear lens in a case of intralenticular foreign body has been reported. It was thought to be due to the presence of intact posterior capsule and a small entry site in the anterior capsule.[6] In small wounds epithelial proliferation creates a plug, which rapidly seals the wound.

We hypothesize that there might have been an equatorial or peripheral anterior capsular injury while administering the injection and the air in the hub of the needle might have permeated into the anterior sub capsular space and produced an unique trauma, which did not result in progression of cataract or visual loss. Intralenticular air can be a rare manifestation of lens injury due to an intravitreal injection.
